# Fine Needle Aspiration in Thyroid Nodules - One Year Experience

**DOI:** 10.3889/oamjms.2015.064

**Published:** 2015-06-05

**Authors:** Irfan Ahmeti, Lliljana Simonovska, Branka Krstevska, Nevena Ristevska

**Affiliations:** 1 *University Clinic of Endocrinology, Diabetes and Metabolic Disorders, Faculty of Medicine, Ss Cyril and Methodius University of Skopje, Skopje, Republic of Macedonia*; 2 *Institute for Lung Diseases and Tuberculosis, Faculty of Medicine, Ss Cyril and Methodius University of Skopje, Skopje, Republic of Macedonia*; 3 *Institute of Patophysiology and Nuclear Medicine “Acad Isak S. Tadzer”, Faculty of Medicine, Ss Cyril and Methodius University of Skopje, Skopje, Republic of Macedonia*

**Keywords:** thyroid nodules, cytological findings, FNA, ultrasound, thyroid carcinoma

## Abstract

**AIM:**

To estimate suspect nodule for benign or malignant characteristics, and to verify cytological features of the node with the fine needle aspiration (FNA) under ultrasound.

**DESIGN:**

A total of 106 patients were analyzed. FNA biopsy was performed at outpatient clinic via ultrasound. Inform consent was signed for each patient. Preparation of procedure with local anaesthesia was made by assistant nurse.

**PROCEDURE:**

Parallel approach of ultrasound guided fine needle aspiration (USGFNA) was used for each patient. This approach allows the operator to observe needle penetration, location and pathway of the entire needle within the neck, thyroid and nodule, which remain visible on the monitor. As a side effect commonly noticed mild pain and dizziness were recorded.

**RESULTS:**

General findings: According the gender, 96 (90.5%) of them were women and 10 (9.5%) men. Median age was 47 ± 9 years. Cytological findings: 5 patients were with papillary carcinoma, 3 with Hurtle cell metaplasia, 1 follicular tumour and 1 with unclear differentiation.

**CONCLUSION:**

Close collaboration between endocrinologists, morphologists and surgeons in a multidisciplinary frame is the key to correct preoperative thyroid cancer diagnosis and optimal treatment. FNA biopsy remains the most accurate diagnostic method in detecting thyroid cancer.

## Introduction

A thyroid nodule represent discrete lesion within the thyroid gland that is radiologically distinct from the surrounding parenchyma [1]. Thyroid nodules are discovered by palpation in 3-7% and by ultrasound (US) in 20-76%. Non palpable nodules detected on ultrasound are named incidentaloma. Nodules are more common in women than in men. Most patients are present with an asymptomatic mass [2]. A survey of clinical members of the American Thyroid Association revealed that most endocrinologists (96%) perform fine needle aspiration FNA biopsy for diagnosis of thyroid nodules [3].

Ultrasound suggested that FNA should be considered for a nodule 1.0 cm or more at the largest diameter if microcalcifications are present and for a nodule 1.5 cm or larger if the nodule is solid or if there are coarse calcifications within the nodule [4]. The American Association of Clinical Endocrinologists recommended FNA even for nodules smaller than 10 mm whenever clinical information or US features arouse suspicion about the presence of a malignancy.

Basic equipment needed to perform FNA biopsy is simple and inexpensive: a 7-14 MHz linear transducer, disposable 10 ml syringes, disposable needled 0.5x15-25 mm, glass slides, alcohol, and gloves. Most thyroid nodules are not malignant, with reported malignancy rates from 3 – 12% [5]. US characteristics are more useful than nodule size for identifying nodules that are likely to be malignant [6]. FNA biopsy (FNAB) of the thyroid gland is an accurate diagnostic test used routinely in the initial evaluation of nodular thyroid disease [7]. Factors associated with an increased risk for thyroid carcinoma are age, < 20 or > 40 years, nodule size > 1 cm diameter, regional adenopathy, presence of distant metastases, prior head or neck irradiation, rapidly growing lesion, hoarseness, progressive dysphagia, or shortness of breath, family history of PTC, family history of MTC or MEN Type 2. High-risk history: History of thyroid cancer in one or more first degree relatives; history of external beam radiation as a child; exposure to ionizing radiation in childhood or adolescence; prior hemithyroidectomy with discovery of thyroid cancer, FDG avidity on PET scanning [8]; MEN2/FMTC-associated RET protooncogene mutation, calcitonin >100 pg/mL. MEN, multiple endocrine neoplasia; FMTC, familial medullary thyroid cancer. Suspicious features: microcalcifications; hypoechoic; increased nodular vascularity; infiltrative margins; taller than wide on transverse view. FNA cytology may be obtained from the abnormal lymph node in lieu of the thyroid nodule (Tab. 1). Sonographic monitoring without biopsy may be an acceptable alternative.

**Table 1:** Reported sensitive features for detection of thyroid cancer on sonography

	Median Sensitivity %	Median specificity %
Microcalcifications	50	85
Absence of halo	66	54
Irregular margins	55	76
Hypoechoic	80	53
Increased intranodular flow	67	81

*Factors suggesting a benign diagnosis include the following:* Family history of autoimmune disease (e.g., Hashimoto), family history of benign thyroid nodule or goiter, *p*resence of thyroid hormonal dysfunction (hypothyroidism, hyperthyroidism), *p*ain or tenderness associated with nodule, soft, smooth, and mobile nodule. The American Thyroid Association (ATA) Guidelines Taskforce on Thyroid Nodules and Differentiated Thyroid Cancer suggests revised management guidelines for patients with thyroid nodules and differentiated thyroid cancer [8, 9].

The aim of this study was to estimate suspect nodule for benign or malignant characteristics, and to verify cytologic features of the node with the fine needle aspiration (FNA) under ultrasound.

## Patients and Methods

In our retrospective study we analyzed 106 patients. In current practice ultrasound (US) with 7-14 MHz linear transducer - 4 cm is used to guide the needle for aspiration nodules. Thyroid lesion with a maximum diameter greater than 1.5 cm or nodule of any size with sonographically suspicious features such as microcalcification, solid feature (vs. cystic), coarse calcifications, texture of the gland, the edges of the nodule and shape (more tall than wide) was an indication for fine needle aspiration. Longitudinal approach was used in all patients. FNA cytology is a minimally invasive and safe, usually performed on an outpatient basis, during the 2013. During the procedure usually 2-4 aspirations are made. Air-dried smears (Giemsa staining) were prepared by using a second glass slide. Frequently 2-3 slides are made for each nodule. Prepared slides were transported to Department of Cytopathology, Medical Faculty, Skopje. After the biopsy has been completed, firm pressure was applied to the punction site. All patients were observed for a few minutes.

## Results

A total of 106 cases of thyroid lesions were studied at the Department of Endocrinology, Medical Faculty, Skopje (Tab. 2). All patients signed inform consent (IC) before intervention of ultrasound guided FNA was made. All of them were informed about the technique of FNA and possible rare risks such as haematoma and pain. Some of the patients (18.87%) complained for local pain and advised to take analgetics. No serious complications such as nerve damage, tissue trauma or vascular injury have been reported.

**Table 2:** Ultrasonographic findings

	Female %	Male %
Patients	96 (90.5%)	10 (9.5%)
Age (median) years	47.5 ± 8.5	46 ±10
Microcalcifications	55	85
Absence of halo	15	54
Irregular margins	35	76
Hypoechoic	60	53

Giemsa staining was used in thyroid cytological preparations. Thyroid FNA classification scheme consists of a four diagnostic categories according to the risk of malignancy: benign lesions, indeterminate lesions according to malignancy, malignant tumours, and non-diagnostic (Tab. 3).

**Table 3:** Implied Risk of Malignancy and Recommended Clinical Management

Cytologic findings of nodules	Risk of Malignancy (n)	Usual Management
Non diagnostic	20	Repeat FNA with ultrasound guidance

Benign	76	Clinical follow-up

Intermediate	3	Repeat FNA

Malignant	5	total thyroidectomy

Indetrminate	2	Lobectomy/subtotal thyroidectomy

Total	106	

Malignant changes often resulted in uni nodular, with no clear limitations and hypoechogenic nodes, with irregularly margins and no clear border, and microcalcifications. Hipoechgenicity is associated with thyroid malignancy [10]. In 6 patients was performed a total thyroidectomy, subtotal in 3 and in 1 lobectomy. In 1 patient total thyroidectomy was made because of PTC in other lobe.

**Figure 1:**
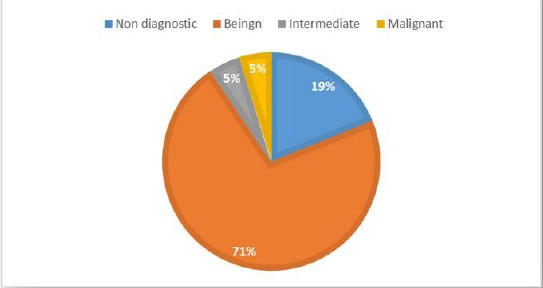
*Cytological findings of ultrasound guided fine needle aspiration (USGFNA)*.

## Discussion

Thyroid nodules are frequent findings when ultrasound is used for evaluation of thyroid gland. They are more common in women than in man. Palpable nodules increase in frequency through the entire life, reaching a prevalence of ca. 4% – 7% of the entire population aged 50 year or older [11]. The prevalence of sonographically detected nodules that cannot be palpated is up to 50%-60% in individuals more than 60 years [12]. Although ultrasound guided fine-needle aspiration cytology is considered to be the reference method for evaluating thyroid nodules, the results are inaccurate in approximately 10-30% of cases. In our study group the percentage of nondiagnostic FNA is 20%. In such cases or in case of indeterminate interpretation FNAB should be repeated. In our group FNA was repeated in 10 patients. In a group of 316 thyroid nodules in 306 patients, adequate cytological specimens were obtained in 97.2% of the nodules in which biopsy was performed, with a 2.8% [13]. The percentage of higher inaccurate results in our patients is because of punction of solid cysts (pure cysts) for evacuation, colloid cysts (acellular, colloid) and not adequately selected from other sonographists which nodule to be FNA performed [1]. Another reason can be the mode of cytological interpretation of pathologist which is not classified according the Bethesda or other consensus guidelines for classification of thyroid cytopathology [14, 15].

In conclusion, close collaboration between endocrinologists, morphologists and surgeons in a multidisciplinary frame is the key to correct preoperative thyroid cancer diagnosis and optimal treatment. Ultrasound FNAB remains the most accurate diagnostic method in detecting thyroid cancer.
